# Understanding extracellular vesicle and nanoparticle heterogeneity: Novel methods and considerations

**DOI:** 10.1002/pmic.202000118

**Published:** 2021-05-03

**Authors:** William Phillips, Eduard Willms, Andrew F. Hill

**Affiliations:** ^1^ Department of Biochemistry and Genetics La Trobe Institute for Molecular Science La Trobe University Bundoora Victoria Australia

**Keywords:** exosomes, extracellular vesicles, microvesicles, nanoparticles

## Abstract

Extracellular vesicles (EVs) are a heterogeneous population of membrane‐enclosed nanoparticles released by cells. They play a role in intercellular communication and are involved in numerous physiological and pathological processes. Cells release subpopulations of EVs with distinct composition and inherent biological function which overlap in size. Current size‐based isolation methods are, therefore, not optimal to discriminate between functional EV subpopulations. In addition, EVs overlap in size with several other biological nanoparticles, such as lipoproteins and viruses. Proteomic analysis has allowed for more detailed study of EV composition, and EV isolation approaches based on this could provide a promising alternative for purification based on size. Elucidating EV heterogeneity and the characteristics and role of EV subpopulations will advance our understanding of EV biology and the role of EVs in health and disease. Here, we discuss current knowledge of EV composition, EV heterogeneity and advances in affinity based EV isolation tools.

**Abbreviations**
AF4: asymmetric flow field fractionation
AFM: atomic force microscopy
Ago2: argonaut‐2
Alix: ALG‐2 interacting protein X
APO: apoptotic bodies
APP: amyloid precursor protein
ARF6: ADP‐ribosylation factor 6
C6S: chondroitin 6 sulfate
CSF: cerebral spinal fluid
ddPCR: digital droplet PCR
EpCAM: epithelial cell adhesion molecule
ERK: extracellular signal‐regulated kinase
ESCRT: endosomal sorting complexes required for transport machinery
EV: extracellular vesicle
EXO: exosome
FCS: fetal calf serum
GFP: green fluorescent protein
GPI: glycosylphosphatidylinositol
HDL: high‐density lipoprotein
HMC‐1: human mast cell 1
HPLC: high performance liquid chromatography
HSP70: heat shock protein 70
IL‐1β: interleukin 1 beta
ILV: intraluminal vesicle
ISEV: international society for extracellular vesicles
LDL: low‐density lipoprotein
L‐EV: large EV
LP: lipoprotein
MHC: major histocompatibility complex
miRNA: micro RNA
MSC: mesenchymal stem cell
MV: microvesicle
MVB: multivesicular body
NCAM: neural cell adhesion molecule
NK: natural killer
NTA: nanoparticle tracking analysis
PEG: polyethylene glycol
RFP: red fluorescent protein
SEC: size exclusion chromatography
SELEX: systematic evolution of ligands by exponential enrichment
S‐EV: small EV
STED: stimulated emission depletion
TEM: transmission electron microscopy
TENPO: track‐etched magnetic nanopore
TFAC: tangential flow for analyte capture
TFF: tangential flow filtration
TNFα: tumour necrosis factor alpha
TRPS: tunable resistive pulse sensing
TSG101: tumour susceptibility gene 101
UC: ultracentrifugation
VLDL: very low‐density lipoprotein
XNA: xenobiotic nucleic acid

## 1 INTRODUCTION

Cells release a variety of biomolecules and nanoscale particles into the extracellular environment as part of normal physiological processes. Extracellular vesicles (EVs) are nanoscale lipid bilayer enclosed particles ranging in size from 20 to 10,000 nm in diameter. EVs are involved in processes such as cell‐to‐cell communication, maintaining cellular homeostasis and the transfer of functional biomolecules [1, 2, 3]. These functions are the result of the cargo EVs carry both on their surface and internally, including proteins, nucleotides and lipids. EV cargo can vary depending on the cell type and differences in cell state leading to differences in EV composition and subsequent function [4, 5, 6]. Cells release a heterogeneous population of EVs of different biotypes such as exosomes (EXOs) and microvesicles (MVs) with varying compositions that result in functionally distinct subpopulations [7]. Changes in physiological conditions and pathophysiological changes due to stimulus or disease can alter the type of cargo associated with EVs and as a result, influence their function. Diversity of EV cargo and associated functionality has led to EVs being of great interest in the potential treatment and early detection of diseases such as cancer and neurodegeneration [8, 9].

### 1.1 EV discovery and characteristics

**FIGURE 1:**
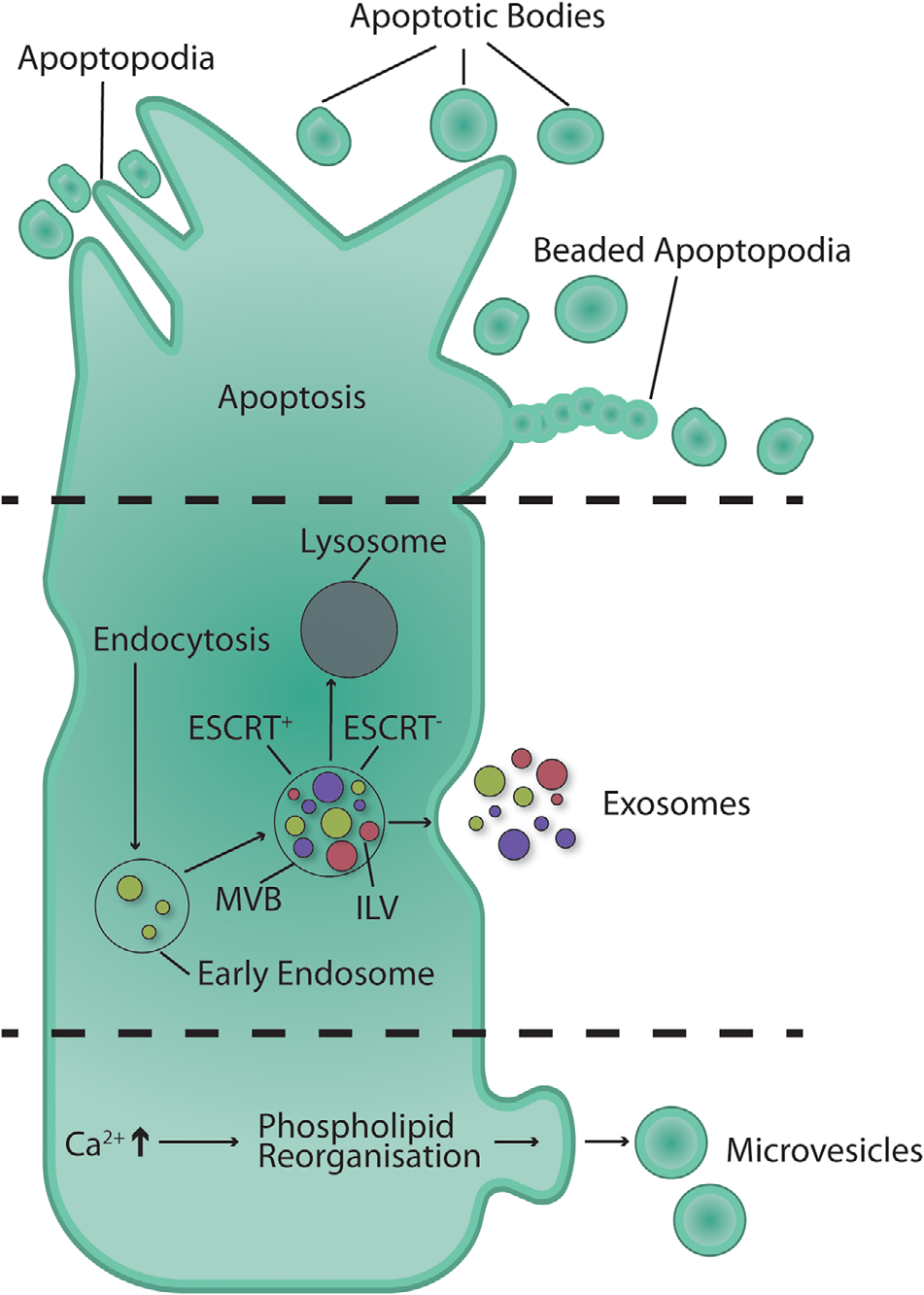
EV biogenesis can be broadly categorized into two main modes of EV release, direct budding from the plasma membrane such as microvesicles (MVs) and apoptotic bodies (APOs) and release of endosomal derived vesicles called exosomes (EXOs). Despite a similar mode of release, there are differences in the release of MVs and APOs. MVs rely on a regulated series of steps begging with an increase in calcium ions, reorganization of the phospholipid membrane, cargo loading and EV release. APOs are the result of regulated cell death and cells exhibit a unique morphology composed of microtubule spikes, beaded apoptopodia and apoptotic bodies resulting from cell disassembly which is distinct from MV release. EXOs are products of the endosomal system, endocytosis generates early endosomes inside the cell, the membrane of the early endosome invaginates creating intraluminal vesicles (ILVs) during which regulated cargo loading through ESCRT+ or ESCRT‐ can take place. The resulting multivesicular body (MVB) may either be degraded by the lysosomes or fuse with the cells plasma membrane release the ILVs into the extracellular space as EXOs

The first recorded use of the term EV was by Aaronson et al. in 1971, although evidence for the existence of EVs can be seen as early as the 1940s by Chargaff et al. [10, 11, 12]. The current interest in EVs was sparked in the early 1980s where EVs were described as transferrin receptor carrying membrane‐enclosed vesicles secreted during reticulocyte maturation [1, 13]. Experiments by Harding et al. used electron microscopy to track secretion of endocytosed gold‐labelled transferrin through the endosomal system and their subsequent release from rat reticulocytes [1]. It was initially thought these vesicles were only relevant as an additional mechanism for preferential loss due to their endolysosomal origin. However, later work by Raposo et al. would demonstrate that B lymphocytes secreted EVs that present major histocompatibility complex (MHC) class II molecules, and provided evidence for a functional role of EVs in antigen presentation and T cell activation [3]. It has since been discovered that EVs can carry a variety of cargo, including proteins, lipids and nucleic acids [4, 5, 14]. These cargoes are thought to dictate the biological function of EVs, which has broadened their originally proposed role of waste removal from cells to cargo delivery into recipient cells, facilitating multiple inter and intracellular processes [13, 15]. EVs have been broadly categorized by their biogenesis pathway into three major EV biotypes: EXOs, MVs and apoptotic bodies (APOs) (Figure 1). EVs can range in size from 20 to 10,000 nm, which can be categorized into two groups, small EVs of a size between 20 and 200 nm (S‐EV) and large EVs of 200+ nm (L‐EV) [16]. The lower bound of EV size is predicted to be between 10 and 20 nm for all phospholipid vesicles as spontaneous vesicle formation below this size is not energetically favourable, but can vary slightly due to differences in membrane thickness and composition [17]. Nomenclature and definition of EVs is an ongoing topic of discussion in the field as biotype and size may not accurately reflect the functional difference of EV populations [18]. The Minimal Information for Studies of Extracellular Vesicles 2018 (MISEV 2018) guidelines are a set of guidelines set out by the International Society for Extracellular Vesicles (ISEV) aimed at improving reproducibility and standardization between studies of EVs [16]. MISEV2018 includes recommendations on nomenclature, including guidelines on the comprehensive analysis of a population's characteristics including size, morphology and cargo. The guidelines acknowledge that characterization efforts are subject to the aims of individual studies and complications that arise from variables such as EV source, isolation method, EV concentration and storage [16]. The guidelines recommend avoiding EV classification based on biotype without confirmation, such as through a Rab27a knockout to inhibit exosome cargo loading or utilizing a compound such as GW4869 to inhibit the formation of EVs through ceramide dependant pathways [16, 19]. In most other cases, the guidelines recommend the use of the terms S‐EV and L‐EV for particles of <200 nm and >200 nm, respectively [16]. ISEV guidelines are a useful framework for ensuring reproducibility of EV studies but do not sufficiently encapsulate the diversity of EV populations brought about by differences in EV composition, origin and function; further development of the guidelines may be required to better represent EV populations and their role within the secretome.

### 1.2 Exosome biogenesis

EXOs are population of S‐EVs that originate from the endosomal system. EXO biogenesis begins with invagination of the parent cell plasma membrane, internalizing transmembrane proteins and forming early endosomes. The early endosome, as it invaginates, creates intraluminal vesicles (ILVs), this structure is subsequently referred to as a multivesicular body (MVB), which can fuse with the cells plasma membrane to release the ILVs into the extracellular space as EXOs [15]. Various proteins are involved in the EXO biogenesis pathway, the GTPases Rab27a and Rab27b are of particular note in transporting the MVB to the plasma membrane [20]. Various pathways of EXO cargo loading and formation have been described, these pathways are responsible for both vesicle formation and cargo loading, including both transmembrane and glycosylphosphatidylinositol (GPI) anchored surface cargo and internal cargo captured in the cytosol during ILV budding [15]. These systems can be categorized as either being endosomal sorting complexes required for transport (ESCRT) dependent or ESCRT independent [21]. ESCRT dependent pathways rely on the ESCRT protein complexes 0, I‐, II‐ and ‐III and associated proteins including tumour susceptibility gene 101 (TSG101), ALG‐2 interacting protein X (Alix) and syntenin [21]. ESCRT 0 is responsible for sequestering ubiquitylated proteins into the endosomal membrane [22]. ESCRT I–II complexes are responsible for membrane deformations into buds containing the sequestered cargo, while ESCRT III performs vesicle scission of the ILVs [21]. Examples of ESCRT independent cargo loading pathways include ILV formation through sphingomyelinase hydrolysis of sphingomyelin to ceramide and the reorganization of tetraspanin microdomains [23, 24].

### 1.3 Microvesicle and apoptotic body biogenesis

MVs are derived from the direct budding of the plasma membrane releasing vesicles into the extracellular space. MV biogenesis is dependent on lipids, proteins and alterations in membrane dynamics and most importantly, the loss of membrane asymmetry [25, 26]. MV biogenesis begins with the mobilization of Ca^2+^, leading to deactivation of flippases and activation floppases, two lipid transporter protein families, in addition to the activation of the enzyme family of scramblases resulting in a loss of membrane asymmetry [26]. The cysteine protease calpain facilitates disruption of the anchorage between the membrane and the cell cytoskeleton; together, these changes facilitate membrane bleb formation [27]. The precise mechanism of MV scission is not well understood, however, it has been suggested that ESCRT proteins may also be involved, as well as ceramide driven budding and ADP‐ribosylation factor 6 (ARF6)‐mediated release [24, 28].

APOs are released by fragmentation of apoptotic cells undergoing programmed cell death. The formation of APOs can be categorized into morphologically distinct stages. These stages include cell rounding, apoptotic membrane blebbing and the formation of elongated cellular ‘beads‐on‐a‐string’ membrane protrusions known as apoptopodia [29, 30]. APOs are an EV population which is less extensively studied, and as a result, less is known about the biogenesis of APOs compared to other EV population such as MVs and EXOs. Biophysical characteristics such as size have been proposed to be between 1 and 5 μm; however, APOs of <1 μm have also been shown to exist [31, 32].

### 1.4 Dissecting the biological functions of EVs

EVs have been shown to play a role in numerous physiological processes such as angiogenesis, cellular migration and cell‐to‐cell signalling [33, 34, 35, 36]. Work by Verweij et al. developed an in vivo zebrafish model utilizing fluorescent reporter CD63‐pHluorin [34]. This model allows CD63^+^ EVs to be tracked in the vascular system of the developing embryo, demonstrating the selective uptake of EVs, their inter‐organ communication capabilities and their ability to provide trophic support during development [34]. While this method provides a useful tool for tracking EVs in vivo it is unable to differentiate between populations of EVs. This exposes the challenge in detecting and tracking different EV populations in vivo due to heterogeneity. Labelling of single markers without functional studies may not be sufficient for identifying EV subpopulations or differentiating between EXOs, MVs and APOs in vivo, future models may be able to address this issue.

The role of EVs in pathophysiological processes underlying diseases such as cancer and neurodegenerative diseases has been more extensively studied. Many different functions for EVs have been described in cancer, including the promotion of tumour formation and angiogenesis [9]. Seminal work by Peinado et al. demonstrated the promotion of pre‐metastatic niche formation by EVs carrying the oncogenic receptor tyrosine kinase MET [37]. Other EV‐mediated mechanisms of metastasis have been established, such as miR‐122 transfer increasing the availability of nutrients within the premetastatic niche of cancer patients with metastatic breast cancer [38, 39]. EVs have also shown to play a role in neurodegenerative diseases, such as transportation of neurotoxic amyloid‐beta in Alzheimer's disease, increase in pro‐inflammatory cargoes such as interleukin 1 beta (IL‐1β) due to neuroinflammation and propagation of the disease‐associated prion protein, PrP^Sc^ which can be found on the surface of EVs derived from prion‐infected cells [40, 41, 42, 43, 44].

The practical applications of EVs are of great interest and are actively explored for the development of therapeutics and diagnostics. Examples of EV therapeutics include the application of mesenchymal stem cell (MSC) derived EVs in regenerative medicine such as myocardial repair after cardiac infarction, utilizing EVs as drug delivery vehicles, or interfering with EV formation [45, 46]. EVs are also of interest in diagnostics, where changes in EV cargo may be indicative of disease before symptoms occur. For example, changes the miRNA carried by EVs, such as a loss of miR‐101 which is associated with an increase in amyloid precursor protein (APP) could be used as an early detection method in Alzheimer's disease [47]. Predictive markers of cancer aggression have also been demonstrated by detecting over‐expression of small heat shock proteins and miRNA known to be associated with metastasis and proliferation [48].

### 1.5 EV populations and heterogeneity

Cells release EV biotypes such as EXOs and MVs, which are derived from unique biogenesis pathways. These EV populations are similar in biophysical characteristics such as size and density, which makes isolation of pure EV populations challenging [49]. Previously EVs were thought to correlate to various size ranges, EXOs frequently cited as being between 20 and 120 nm, MVs from 50 to 1000 nm and APOs 50 to 2000 nm [50, 51]. However, growing evidence suggests that these size ranges do not represent pure biotype populations and are difficult to verify due to the lack of biotype specific markers [52, 53, 54]. This makes EV purity in terms of individual functional populations difficult as physical characteristics such as size or density may not correlate to functionally distinct subpopulations. Previously assumed homogenous EV populations have been shown to contain distinct functional subpopulations with unique composition through density gradient isolation and proteomic analysis of the collected fractions using in gel preparation and digestion, LC‐MS/MS spectrometry and label‐free quantification [6, 55]. Gene expression differences in the heart endothelial cell line H5V incubated with either EVs from low‐density or high‐density gradient fractions have shown functional differences within populations of heterogenous EVs [55]. The heterogeneity of EVs outlines the need to isolate and characterize functionally distinct EV subpopulations, as EV function is primarily the result of the EV composition [56]. Composition offers a relevant alternative for isolating and characterizing functionally distinct populations of EVs. Also, biological fluids such as blood will contain EVs originating from many different cell types as well as lipoprotein particles (LPs) which introduces greater complexity to EV and total nanoparticle populations [57].

### 1.6 Isolating EV subpopulations

To investigate the functions of EVs, it is necessary to separate them from other particles and molecules which if not removed could lead to misattribution of biological processes to EVs. The purity and specificity of isolation methods may differ depending on the downstream application of the isolated EVs. For example, when searching for biomarkers in a diagnostic setting, broad isolation is more useful to increase the chances of finding a relevant disease marker. Conversely, a targeted isolation method capable of isolating specific EVs, such as by the presence of particular membrane proteins, is required when investigating the biological function of a functional EV subpopulation. EV purity can therefore be defined in terms of the absence or presence of contaminants, or purity in terms of the target EV subpopulation to be isolated. EV purity in terms of contaminants is the presence of proteins, protein aggregates and LPs. LPs are a well‐characterized mono‐layered particle of similar size range to EVs. Purity can also be considered in terms of EV subpopulations as EVs comprised diverse populations of particles with overlaps in size, density and composition across EV biotypes and cell types.

Even among a single cell type, EVs can have a variety of subpopulations that can change due to cell conditions, clonal drift and duration of culture before isolation. De Jong et al. demonstrated the effect of varying cellular conditions on EV cargo profiles by exposing HMEC‐1 epithelial cells to different types of stress such as hypoxia, hyperglycaemia and inflammation [58]. The study found EV protein and mRNA expression changes in hypoxic and tumour necrosis factor alpha (TNF‐α) treated cells were distinguishable from the control cells [58]. At the same time, there were few biophysical differences between EVs from treated and non‐treated groups [58]. This highlights the constraint of biophysical characteristics for isolation, which is incapable of separating subpopulations that differ based on their cargo. The purpose of this review is to discuss EV heterogeneity and present a case for composition‐based isolation which may provide a superior method to understanding functional EV subpopulations that will lead to greater insights into EV biology.

## 2 EXTRACELLULAR VESICLE HETEROGENEITY

**FIGURE 2:**
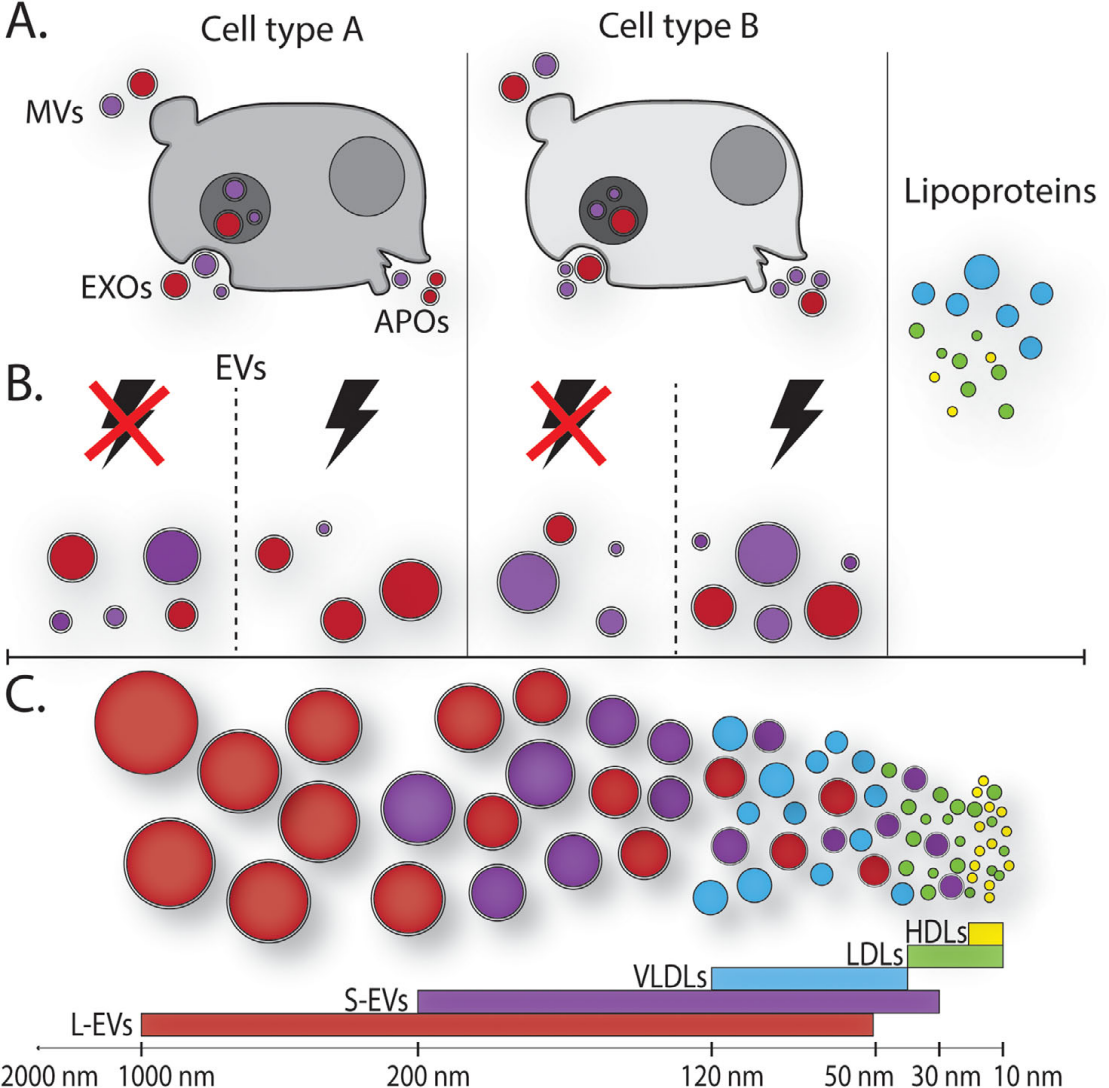
EV populations are complex and heterogeneous which can exist on multiple levels. (A) The release of extracellular vesicles (EVs) including microvesicles (MVs), apoptotic bodies (APOs) and exosomes (EXOs). (B) Variable release of EVs into the extracellular space depending on cell type, cell state and environmental stimulus. (C) Lipoproteins (LPs) that overlap in terms of size with EVs including high‐density lipoprotein (HDL), low‐density lipoprotein (LDL), very low‐density lipoprotein (VLDL). A lack of biotype specific markers for EVs leads to EVs being categorized into two size groups, S‐EVs and L‐EVs. The overlapping size of small EVs (S‐EVs), large EVs (L‐EVs) and non‐EV particles presents a significant challenge for isolating EV subpopulations

Cells release EVs with different biophysical characteristics such as size, density, charge and varying cargo composition into their extracellular environment [6, 51, 53, 54, 55, 59]. This results in a heterogeneous population of EVs. Mass spectrometry based proteomics, and nanoparticle tracking analysis (NTA) size analysis, has demonstrated that size and compositional heterogeneity extends to EVs derived from different cell types. For instance, it has been shown using label‐free quantification proteomics based on in gel digestion, MS/MS and data‐independent acquisition that MSC EVs share common proteins between glioblastoma and hepatocellular carcinoma EVs [60]. Still, EVs from each cell type also carry unique proteins not found in EVs from the other cell types [60]. Research utilizing asymmetric flow field flow fractionation (AF4) a highly accurate size‐based isolation method also demonstrated differing EV associated protein and miRNA signatures from B16‐F10 melanoma, 4T1 breast cancer and Pan02 pancreatic cell lines [51]. EV heterogeneity also extends to unique and shared proteins between S‐EV and L‐EV populations, with S‐EVs being more likely to carry proteins that could predict the cell type of parental cell of the EV [60]. However, EV cargo has not been shown to reflect an EVs biotype, as protein markers thought to be associated with exosomes can be found in both S‐EVs and L‐EVs [60]. As illustrated in Figure 2, heterogeneity can play a role at multiple levels: (i) differences in EV subpopulations secreted by individual cells and cells of the same type, (ii) presence of EVs derived from different cell types/cell sources, and (iii) overlap with other nanoparticles such as LPs.

Cells respond to stimulus and changes in the extracellular environment such as temperature, physical stimulation and toxins. These adaptive responses can include survival responses, such as increased transcription of heat shock proteins in response to changes in temperature [61]. Adaptive responses by cells also involve changes in EV secretion, this mainly involves modifications to the cargo loaded into EVs but can also include changes in the biophysical characteristics of the secreted EVs such as size [48, 62]. For example, EVs secreted by hypoxic glioblastomas have been found to carry proteins associated with hypoxic response, and in cancer can aid in tumour growth by increasing tube formation. Interestingly, this increased angiogenic behaviour has also been found in hypoxic MSC‐derived EVs [62, 63]. This variable response by individual cells could result in diverse, heterogenic populations of EVs originating from the same cell type.

Multi‐cellular organisms require a range of specialized cell types to survive. There is contention on the number of human cell types, but based on histology it is usually cited there are approximately 200 human cell types [64]. Recently, projects such as the Human Cell Atlas have been using single cell approaches to answer this question more accurately [65]. It is however difficult to define cell types due to issues such as non‐specific markers as well as heterogeneity of cell states [65]. It is known that specialized cells such as epithelial cells, neurons and lymphocytes release different populations of EVs with various functions such as platelet‐derived EVs exhibiting procoagulant properties, dendritic cell‐derived EVs involved in T‐cell activation, and microglial EVs modulating neurotransmission through sphingolipid metabolism [66, 67, 68]. EVs may carry proteins associated with their origin cell type (Table 1), such as CD61 for platelets or CD90 for MSCs [69, 70]. While these markers may be tissue or cell type associated, they may not be exclusive to those tissues, neural cell adhesion molecule (NCAM), for example, is considered a neuronal marker but may also be found in endocrine and muscle tissues [71]. The lack of a definitive cell‐specific EV markers and the variability of EVs from different cell types contributes to further EV heterogeneity in more complex environments such as biofluids or tissue samples making attribution of an EV to a specific cell type difficult.

**TABLE 1:** Proteins associated with particular cell types, these proteins are generally more abundant in these particular cell types, making them more likely to be EV associated and useful for isolation or characterization of EVs derived from these cells

Cell type	Associated protein	Reference
Neural	CRABP1, CD56 (NCAM)	[72]
Microglia	Anandamide (ANA), CD14	[73, 74]
Hepatocytes	CD29	[75]
Mesenchymal stem cells	CD90, CD29, CD44, CD73	[70]
Platelets	CD61	[76]
Epithelial	EpCAM, TSPAN8	[77, 78]
Endothelial cells	CD31	[79]
Fibroblasts	Integrin α6	[80]

EV isolation from biofluids such as serum, plasma, cerebral spinal fluid (CSF) and urine, present further challenges due to the presence of extracellular protein and other similarly sized nanoparticles including cellular debris, aggregates, viruses and LPs [81, 82, 83]. These particles overlap in size and density to EVs and originate from multiple cell and tissue types representing a significant challenge for targeted EV isolation by contributing another layer of heterogeneity [50, 84]. Additionally, non‐EV particles are involved in cell‐to‐cell communication, such as soluble cytokines with pro/anti‐inflammatory properties or oxidized low‐density lipoprotein (LDL) capable of altering macrophage gene expression [85]. LPs are of particular note due to their physical similarities to EVs and their relative abundance in blood compared to EVs. LDLs vastly outnumber EVs alone at 10^15^ LDL particles per mL, with the total concentration of LPs estimated at 10^16^ particles per mL [86]. The concentration of EVs in plasma is not well defined due to variability in isolation methods. A literature survey by Johnsen et al. proposes an upper EV concentration of 10^10^ EVs per mL; however, as noted by the authors, this count may include contaminating particles [86, 87]. As LPs vastly outnumber EVs in plasma it is necessary to separate EVs from contaminating LPs as well as from other nanoparticles and extracellular proteins, particularly for functional studies where the signalling capability of these biomolecules and nanoparticles could confuse research results.

## 3 LIMITATIONS OF SIZE‐BASED EV ISOLATION

EV isolation most commonly relies on size‐based techniques such as ultracentrifugation (UC) and size exclusion chromatography (SEC). These methods appear precise and selective but rely on an underlying assumption of a correlation between EV size and biotype. Questions remain about the correlation between EV size and composition; it is currently not completely understood whether EV certain biogenesis pathways are more likely to produce vesicles of a particular size. EXOs are physically limited in maximum size by the MVB (250–1000 nm) from which they originate, Edgar et al. reported that ILVs, which upon release become exosomes, are generally approximately 150 nm in diameter [88]. The limits of membrane‐derived EVs in terms of size, and the effects of cargo loading on size remain unclear. EV isolation based on size may therefore not represent a targeted isolation method for resolving EV heterogeneity. Alternatively, isolation methods that do not rely on size such as polyethylene glycol (PEG) precipitation may negatively affect EVs, this has been seen in atomic force microscopy (AFM) experiments where EVs isolated with PEG likely retained residues on their surface which may artificially increase particle size, this also raises questions as to whether the PEG remnants may interfere with the binding of ligands to EV surface receptors [89]. Size‐based isolation UC‐based methods, SEC, tangential flow fractionation (TFF) and AF4 all rely on size to isolate EVs. UC depends on the sedimentation of particles under centrifugal force, where lower speed centrifugation is conducted to remove larger particles, followed by high‐speed centrifugal forces to pellet smaller particles. Yield can differ depending on rotor selection and duration of centrifugation, with longer durations allowing more time for smaller particles to sediment [90]. UC cannot selectively isolate particular subpopulations and suffers from user variability due to sample loss during pellet collection and from pipetting error affecting reproducibility as well as co‐isolation of undesired particles of similar size including LPs [49, 91]. Furthermore, EVs from cardiomyocyte progenitor cells isolated by SEC has been shown to induce more extracellular signal‐regulated kinase (ERK) 1/2 phosphorylation in microvascular endothelial cells than UC isolated EVs [92]. This showed EVs isolated from SEC have higher functionality compared to UC isolated EVs, it was suggested that this is due to higher shear forces experienced by UC EVs damaging vesicles [92].

Recently, interest has increased in SEC based methods; these are usually column based utilizing a resin that separates particles of the desired size range. This is accomplished by porous resin that separates particles by size as they travel through the column, resulting in larger particles incapable of entering the pores eluting earlier than smaller particles that are slowed down by the pores, leading to differing elution times for smaller and larger particles. These columns can be operated with or without the use of high‐performance liquid chromatography (HPLC) equipment which utilizes pressure as opposed to gravity, the latter of which does not require specialized equipment, HPLC allows for better reproducibility and isolation speed compared to manual gravity collection. However, while quick, SEC suffers from resolution issues when compared to more advanced methods like AF4. This can be improved with resins that work over different size ranges and resins that incorporate additional binding techniques increasing the versatility of SEC based isolation. Examples such as charge based SEC resins or bind‐elute systems, comprising a bind‐elute resin containing beads with a positively charged hydrophobic core, which capture molecules of a size capable of entering the core such as extracellular protein, which aims to improve purity but may not always improve resolution [93].

Another size‐based EV isolation method includes TFF which utilizes a porous membrane and pressure to exclude particles below a specific size cut off while retaining and concentrating larger particles. TFF is typically coupled with a polishing step such as ion‐exchange chromatography (IEX), affinity isolation, SEC and density gradient isolation due to the retention of contaminants [94]. Recently, more advanced techniques utilizing microfluidic devices have become more prevalent. One such example utilizes pores on an ultrathin membrane and TFF as the capture method, this operates in a capture, clean, and release mode called tangential flow for analyte capture (TFAC) which may overcome some of the contamination issues of larger and smaller particles [95]. One method for isolating EVs based on size is AF4. AF4 has recently gained interest as a powerful high‐resolution method of isolating specific EV size populations [51]. AF4 while a powerful tool suffers from low yield due to a small working volume. While capable of providing accurate isolations of various particle size populations yield can often be lacking and may not be practical or affordable for all studies.

### 3.1 Lipoproteins

LPs are a group of lipid monolayer nanoparticles of similar size range to EVs. LPs are most commonly associated with the blood but can also be found in CSF [83]. LPs are categorized based on their density and include chylomicrons, very‐low‐density lipoproteins (VLDLs), LDLs and high‐density lipoproteins (HDLs) [96]. The primary function of lipoproteins is to transport fats such as phospholipids and triglycerides [96]. Importantly, they have functionality beyond fat transport, which likely includes cell‐to‐cell communication through LDL receptors as well as HDLs carrying miRNA [27, 28, 29]. In addition to triglycerides, phospholipids, cholesterol esters and free cholesterol, the other primary component of lipoproteins is apoproteins [97]. These surface proteins are essential for lipoprotein structure, thus a vital component of apolipoproteins. Particular apoproteins are associated with different lipoproteins, in the case of LDL apolipoprotein apoB100 is a useful quantitative marker as there is only a single apoprotein b molecule per LDL particle [98]. HDLs are more complex and carry a variety of proteins but are generally characterized by the presence of ApoA1 [99]. HDLs have been shown to transport a variety of proteins and miRNA, the latter of which may adversely affect EV small RNA studies [91].

LPs are a significant constituent of plasma, vastly outnumbering EVs and therefore are a significant source of contamination in EV isolations [100]. This is due to size overlaps with EVs, HDLs range from 8 to 16 nm, LDLs from 10 to 40 nm and VLDLs from 40 to 120 nm [96]. This complicates EV isolations from plasma as isolated populations include lipoproteins within a similar size range. This is further complicated by density overlap between EVs and HDLs [101]. Few particle detection methods can tell the difference between EVs and other nanoparticles. Consideration for lipoproteins in vitro must be considered if fetal calf serum (FCS) is utilized due to the presence of HDLs. This is true even in EV depleted FCS as the ultracentrifugation protocol commonly used to deplete EVs is often insufficient to remove all particles leading to contamination, particularly concerning HDL associated miRNA [102]. Due to the overlap of LPs of a similar size as smaller EV populations, suitable isolation strategies are required.

### 3.2 Removing lipoproteins from EV plasma isolations

Separation of contaminating LPs is vital for functionality studies to remove any bias that may be due to signalling capabilities of LPs as well as ensuring accuracy of quantification and normalization of cargo to number of EVs for experiments and therapeutics requiring dosage. Size‐based methods are incapable of separating EVs from all LP due to similarities in size and density [57, 86]. Density gradients have been widely used for EV subpopulation studies as they can further separate EVs based on density and are used typically following a size‐based isolation step [59, 103, 104, 105]. Density gradients are capable of removing LPs due to their density differences to EVs as well as proteins [86, 101]. However, density gradients cannot remove HDL as they share a density overlap with EVs. Additionally, the procedure is time‐consuming and reliant on access to an ultra‐centrifuge [57].

Recently, dual‐mode chromatography systems have been utilized to deplete LPs from plasma [106]. By exploiting the charge difference between EVs and LDLs it is possible to remove LDLs by using their positive charge through ion‐exchange chromatography, this is successful in removing a large number of LDL particles as demonstrated by the considerable reduction of apoB100 [106]. The difficulty is encountered in this method as IEX cannot remove HDLs as they share charge with EVs. Therefore, SEC is used to separate HDLs based on size; however, SEC resins lack the resolution to remove HDLs completely but is sufficient for the removal of a majority of non‐EV particles including HDLs and non‐vesicular protein [106].

Additionally, when removing HDLs by SEC unless a resin with a smaller isolation range is utilized; otherwise, low resin resolution leads to a significant amount of HDL remaining [106]. While the increase in EV purity from this method is useful, it may not be enough for functional assays where LPs may still interfere. Alternative isolation methods may include the use of heparin sulphate or chondroitin 6‐sulfate (C6S) as well as specific apoprotein antibodies to capture LPs and deplete them directly [107].

## 4 EV COMPOSITION‐BASED STRATEGIES FOR THE STUDY OF EV HETEROGENEITY

The aforementioned section outlined the limitations of size‐based isolation for the study of EVs. The heterogeneous nature of EV isolates containing EVs derived from different cell types and states as well as contaminants with overlapping size highlight the need for more targeted isolation approaches. The development of these technologies is particularly relevant for the study of EVs derived from complex biofluids containing impurities as well as EVs derived from multiple cell types. Composition‐based isolation will also enable the isolation of EV subpopulations with overlapping size but distinct composition from individual cell types. This will in turn advance the study of the biological functions mediated by EV subpopulations.

### 4.1 EV composition and function

Although unique markers for distinct EV biotypes such as EXOs have not been identified, various EV‐associated cargoes have been documented that can be used to identify EV subpopulations. These markers consist of proteins present both on the vesicular surface, such as the transmembrane proteins CD63 and CD81, as well as proteins in the vesicular cytoplasm, such as Alix, flotillin and syntenin [31, 108]. Currently proteomic approaches, most commonly label free, are used to characterize the composition of EV isolates and different EV populations [109]. Metabolic labelling based quantification such as stable isotope labelling of amino acids in cell culture (SILAC) can provide extremely accurate data about EV composition in cell culture models and is capable of being extended to mouse models, providing a powerful tool for resolution of EV s associated proteins [110, 111].

Proteins present on the EV surface are the most suitable targets for composition‐based isolation as they are readily accessible and not obstructed by the EV lipid bilayer. EV surface proteins are relevant for EV uptake, and therefore, EV‐mediated cell‐to‐cell communication [78, 112]. Examples of EV surface proteins used for characterization and isolation include the tetraspanins CD9, CD63 and CD81, which can be differentially expressed between different EVs [6]. Proteomics conducted on human primary monocyte‐derived dendritic cell EVs isolated with ultracentrifugation followed by affinity isolation with CD9, CD63 or CD81 antibodies found that many S‐EV related markers and exosome markers are present in EVs of various sizes, for example, CD63 was found in both S‐EV and L‐EV populations, this provides further evidence for the heterogeneity of EVs [6]. Tetraspanins also influence EV function; this can range from cellular targeting, impacting selective cargo loading due to changes in the localization of tetraspanins on the plasma membrane and a direct role in communication through cell surface receptor signalling [23, 113]. Differences in biodistribution of EV subpopulations comprised different tetraspanins has also been demonstrated through luciferase tagged CD63 and CD9 in mice [114]. CD63 was found to be enriched in the brain and kidneys compared to CD9 EVs which were found to be enriched in the GI tract and lungs [114].

EV‐associated tetraspanins are also involved in disease progression, in a study of a rat pancreatic adenocarcinoma cell line with a knockdown of CD151 and Tspan8 it was demonstrated that tetraspanins are directly involved in cancer metastasis and facilitate cross‐talk with surrounding cells, facilitation of epithelial–mesenchymal transition and ultimately tumour progression [78]. This was shown through knockdown of CD151 and Tspan8 cell‐derived EVs where wildtype EVs increased metastasis in rats with the knockdown cells, implicating both Tspan8 and CD151 in tumour metastasis [78]. CD151 and Tspan8 EVs were also shown to influence inflammatory phenotypes and induce stroma cell activation versus knockdown cell‐derived EVs [78]. Differences in tetraspanin expression can also change cell tropism of EVs, EVs with differing Tspan8 and CD104 expression have been shown to bind preferentially to cells, Tspan8 EVs were shown to bind to lymph node stromal cells and Tspan8 to cells expressing CD11b, CD18 or CD54 [56].

Further research is required on how EVs are targeted to their recipient cells beyond the role of tetraspanins. Still, it likely involves the combined composition of different surface proteins and lipids associated with EVs [45]. Another possible mechanism of cell tropism of EVs is through differential integrin expression. Evidence for the role of integrins comes from work on EVs isolated from the human pancreatic cell lines BxPC‐3 or HPAF‐II that are known to metastasise to the liver as well as human breast cell lines MDA‐MB‐231 and MDA‐MB‐468 that are known to metastasise to the lung and both liver and lung respectively. The isolated EVs were fluorescently labelled and retro‐orbitally injected into mice [115]. The labelled EVs were found to distribute to their respective organs after 24 and were taken up by resident cells [115]. Quantitative in solution digested LC–MS/MS proteomic analysis using data dependant acquisition of isolated EVs found particular integrin signatures associated with organotropism, for example, integrin alpha 6, integrin beta 4 and integrin beta 1 was present in lung‐trophic EVs and integrin beta 5 and integrin alpha v was associated with liver‐trophic EVs [115]. A knockdown of integrin beta 4 reduced the lung metastatic ability of the lung‐trophic MDA‐MB‐231 sub‐line 4175‐LuT as determined by bioluminescence imaging, the metastatic ability was then able to be rescued by non‐knockdown 4175‐LuT EVs [115]. This example highlights the ability of integrins to dictate cancer metastasis which could indicate a role for integrins in organotropism of EVs [115]. Knowledge on the composition of EV associated membrane proteins can be used to inform isolation strategies, targeting EVs based on markers that indicate the function or specific cell targeting. However, there is still a need for general EV markers to isolate and characterize EVs. The lipid raft protein stomatin has been proposed as a universal EV specific marker, having been detected from epithelial cell‐conditioned media and EVs isolated from biofluid [116].

Internal markers are typically used for characterization and may contribute to EV function following EV uptake and cargo release into the recipient cell. Internal protein cargo commonly found in EVs includes Flotillin‐1, Alix, TSG‐101 and Syntenin [6]. Internal cargoes can vary depending on the environmental and stimulatory factors affecting the parental cell [7]. For example, EVs of differing internal cargo composition and loading mechanisms have been demonstrated, including neuroinflammatory markers from CSF isolated EVs in HIV^+^ individuals and preferential nSMase1 dependant packing of PrP^Sc^ in prion‐infected cells [43, 44].

Small RNA is carried by EVs and has been a research focus particularly concerning EV associated microRNA (miRNA) for diagnostics [117]. Ribonucleoprotein such as argonaut‐2 (Ago2) can carry small RNA and may be associated with EVs, presenting a possible marker to distinguish populations of small RNA carrying EVs [118, 119]. Whether EVs carry miRNA is contentious, quantitative analysis using digital droplet PCR (ddPCR) has shown low copy numbers of miRNA carried by EVs isolated by ultracentrifugation, this may suggest the existence of miRNA enriched populations that cannot be sufficiently isolated by size from the rest of the EV population [120]. However, there is further debate on whether extracellular miRNA is not associated with EVs but rather may be associated with extracellular Ago2 [91]. Improved EV isolation methods could provide a useful tool to address this question by enabling isolation of miRNA enriched EV subpopulations. This will contribute to improving the development of miRNA based diagnostics [121].

Despite the large body of knowledge surrounding EV function not much is still known about the precise mechanics of EV uptake and cargo release, this is in part due to the difficulties in detecting and tracking EV cargo release in vivo. One in vivo study examined metastasis using a Cre‐*lox*P system where Cre^+^ cells secrete EVs containing either Cre mRNA or Cre protein, a reporter cell line expressing red fluorescent protein (RFP) changes to Green fluorescent protein (GFP) when Cre‐mediated recombination occurs [122, 123]. The study looked at the uptake of EVs by tumour cells in mice and demonstrated EV uptake by tumour cells and the ability of mRNA carried by tumour EVs to increase metastatic behaviour [123]. However as noted by the authors, Cre protein was not detectable in Cre^+^ EVs, whereas Cre mRNA could be detected reliably [122]. This system can be used to detect EV uptake but may not be reliable for detecting cargo release, a current challenge in the field. A recent example of this problem can be found in a study utilizing a CRISPR‐CAS9 reporter system for EV‐mediated RNA transfer termed CROSS‐FIRE [124]. The study demonstrated that while EVs may be taken up readily, RNA transfer efficiency may be different for various cell types and was overall quite low [124]. This phenomenon can also be seen in multiple EV CRE recombinase systems, with low delivery and few positive reporter cells [122, 123, 125]. This phenomenon in EV cargo delivery may be due to EVs being degraded by the endosomal system following uptake, but could also be indicative of specialized EV sub‐populations. Alternative methods of EV signalling have been demonstrated that draw similarities from viruses by surfing on filopodia resulting in transport directly to the endoplasmic reticulum [126]. This may circumnavigate endosomal degeneration and could result in a higher potency of the therapeutic cargo. Further research should focus on the specific features of the subpopulation EVs able to successfully deliver their cargo and isolation of these specific subpopulations [127].

### 4.2 Affinity based isolation strategies

Affinity based isolation methods typically involve the targeting of EV surface proteins such as CD81, CD63 or CD9 [128, 129, 130]. Combinations of lectin isolation plus CD81, CD63 and CD9 conjugated‐antibody bead capture have been utilized to examine size and lectin profile differences of urine EVs to better inform EV isolation targets [131]. This approach has been used to isolate EVs carrying the transmembrane antigen glycoprotein A33 from the LIM1215 colorectal carcinoma cell line with microbeads [130]. Neuronal associated NCAM, and L1CAM‐enriched EVs have been isolated using a combination of polymer EV isolation and immunoprecipitation from peripheral blood [132]. Tumour‐derived EVs have been isolated from patient tissue samples using hear shock protein 70 (HSP70) specific aptamers [133]. These studies demonstrate the ability of affinity‐based capture to target particular populations of EVs for research as well as diagnostic purposes. Before affinity isolation samples may undergo an initial isolation step to remove unwanted impurities, contaminants and aggregates that might interfere with binding. Other pre‐treatment methods such as ultrafiltration or tangential flow filtration can increase concentration and remove smaller particles such as protein while retaining larger EVs.

#### 4.2.1 Bead‐based isolation

Typically, magnetic beads are used for antibody isolation [6, 59, 128]. This involves incubating a biotinylated antibody with the sample and applying magnetic streptavidin beads. The resulting magnetic complex containing the target of interest is subsequently retained using a magnet and the supernatant removed. This method has been used to benchmark EV isolations *in vitro* and can also be utilized in resin‐based columns [128, 129].

Multiplexed bead‐based systems for the characterization and isolation of EVs have been demonstrated previously. A comparison of natural killer (NK) and platelet‐derived EVs using fluorescent probes was used for characterization and screening of potential subpopulation differences of tetraspanins between the two cell types. A panel of 36 antibodies was used to isolate NK and platelet‐derived EVs, the populations were counted using fluorescent probes, flow cytometry and stimulated emission depletion (STED) super‐resolution microscopy. This study demonstrated different tetraspanin composition between NK‐ and platelet‐derived EVs, with cell type associated markers being present on their relevant cells such as CD2, CD8 and CD56 for NK cells and CD41b, CD42a and CD61 for platelet EVs [134]. This method was capable of isolating EVs of different surface compositions, demonstrating the ability of affinity‐based approaches to identify, isolate and then characterize EV subpopulations based on differences in EV surface composition.

#### 4.2.2 Chromatography‐based isolation

HPLC can be used to isolate EVs using affinity resins that antibody bind to, this has the benefits of an HPLC system including reproducibility and capability of running in series with other columns such as IEX to remove contaminants. Monoliths are single structure highly permeable support for liquid chromatography. Monoliths have the advantage of larger gaps between the matrix which facilities higher flow rates and may also help avoid damage of EVs as their laminar flow reduces shear forces introduced by turbulence [76]. Monoliths have been used previously to isolate CD61^+^ particles from blood plasma with only a brief centrifuge spin, resulting in fast, pure isolation. This study demonstrated the presence of EV associated markers and particle size range expected of S‐EVs [76, p. 61]. However, despite this, the authors noted that elution conditions might affect epitope binding efficacy and additional concentration may be required due to the isolate being diluted [76, p. 61]. Further validation is needed, but the method has shown promise for fast affinity isolation from complex biofluid [76]. This highlights the general drawbacks to antibody based approaches, the need for surface markers and compatible antibodies.

#### 4.2.3 Aptamers

Aptamers are nucleotide sequences such as DNA and RNA that can fold into a 3D structure capable of binding to a specific ligand [135]. Aptamers have been raised against targets such as CD133 and Epithelial cell adhesion molecule (EpCAM) for EV capture and imaging using fluorescently tagged aptamers [136]. Aptamers have been proposed in various formats and have been demonstrated to be capable of binding to a variety of proteins including HSP70, CD63 and thrombin [137, 138, 139]. Additionally, aptamers can be modified to include features such as fluorescent tags and biotin tags. The advantages of aptamers primarily lie in their high‐specificity, low immunogenicity and cheaper cost to produce compared to antibodies [135]. Aptamers are selected through systematic evolution of ligands by exponential enrichment (SELEX) which results in highly specific targeted aptamers [138]. For EV isolation, aptamers have been utilized to isolate cancer‐related HSP70 positive EVs from both blood and urine, providing another tool for EV isolation and quantification [135]. The binding strength and stability of aptamers are weak compared to antibodies; however, analogue xenobiotic nucleic acids (XNA) may improve the binding strength and stability of aptamers for isolation, conversely, the weak binding strength of aptamers could be viewed as an advantage allowing for easier elution under less harsh conditions [140, 141]. Finally, eluting EVs from aptamers can utilize either enzyme such as DNase/RNase or an increase in magnesium, which is less aggressive and damaging to EVs than large pH changes required by antibodies which may degrade EVs [135].

#### 4.2.4 Microfluidics

Microfluidics has begun to be utilized more in EV research, and while most of the interest lies in developing diagnostic devices, some approaches have been established to isolate EVs [142]. Microfluidic devices focusing on characterization have been utilized to quantitatively identify the presence of surface proteins using digital PCR in EV populations grouped into emulsified oil droplets, defining smaller groups of EVs by their surface signature to a single EV level [143]. Other microfluidic devices for detecting composition have been proposed; these include charge based, immunofluorescence and surface plasmon resonance devices [144].

EV isolation methods have also been proposed utilizing microfluidics, this includes affinity capture using conventional antibodies but can also be coupled with nanoscale topographies such as herringbone grooves, capable filtering particles of a particular size which can then be further selected based on affinity [145]. Additional topographies include nanoscale pores that can trap EVs based on size and elute the captured EVs following a wash step [146]. Affinity isolation methods have also been described such as system track‐etched magnetic nanopore (TENPO) which utilizes magnetic nanopores and magnetic labelling to isolate EVs based on affinity without prior sample processing [147]. Further advantages to microfluidics include the capability to implement an entire process on‐chip; this could consist of EV lysis and PCR and has relevance for liquid biopsies [148]. These methods may be relevant for isolating individual EVs based on size or other biophysical attributes. However, microfluidics can suffer from poor scaling, clogging and low throughput due to the scale of the devices.

## 5 SUMMARY AND CONCLUSIONS

EVs are membrane‐enclosed nanoparticles that have demonstrated roles in physiological processed such as cell‐to‐cell signalling, and pathophysiological roles such as neurodegeneration and cancer. Understanding the role of EV cargoes in their functions is important for furthering the field and providing insight into basic biology that can have implications for the development of therapeutics and diagnostics. Additionally, engineered EVs offer a method of therapeutic delivery by selectively loading therapeutics into EVs that can then be targeted to particular cell types by altering membrane proteins and lipid content [149]. However, further understanding of the biological processes underlying EV targeting, cargo loading, uptake, release and potency is required before it can be exploited fully.

EV heterogeneity presents a significant challenge in EV isolation and characterization, particularly from biofluids. The overall complexity of biofluids not only from constituent proteins and cell debris but from heterogeneous nanoparticle populations makes a targeted isolation method a necessity to understand the signalling carried out be EV subpopulations. By isolating EVs based on composition, it is possible to isolate relatively pure populations that are more relevant for studying EV subpopulations and their functions [78]. Proteomics is a valuable technique in resolving differences in composition of EV subpopulations and can be used to inform targets for affinity isolation, as well as quantitative proteomics for comparison between EV populations [6, 115]. However, EV isolation methods should be chosen based on the experiments being conducted. For example, in biomarker discovery, the origin of the marker is often not immediately relevant as the main consideration is the detectability of the marker of interest.

Furthermore, considerations to the effects of the isolation method on the isolated EVs need to be considered, such as aggregation, coating, damaging or altering EVs. For example, in affinity isolation surface proteins may lose their binding capabilities following elution of the antibody due to the harsh conditions of elution as well as damage EVs. Alternative affinity ligands such as aptamers may represent a way around this issue as they can be degraded under gentler conditions compared to antibody elution.

Despite the heterogeneity of EVs, biophysical properties are still an important part of EV characterization. Particle size and count is an integral part of EV characterization. NTA is the most popular method of determining particle size and is based on the Brownian motion of particles [150]. The resolution of NTA is limited to approximately 60 nm; however, this can be improved through the use of fluorescence NTA [151]. Other methods include tunable resistive pulse sensing (TRPS) which is based on measured differences in electrophoretic mobility of EVs passing through a nanopore [152]. Despite ongoing discussion regarding the accuracy of these methods, both are unable to distinguish between EV biotypes or LP contaminants which may lead to inaccurate population counts.

EV composition can be used in conjunction with the aforementioned characterization techniques. By targeting EV cargo it is possible to understand their function better, this can be with existing methods such as fluorescent NTA, flow cytometry or scanning electron microscopy. Morphology is another point of differentiation for EVs [153]. Transmission electron microscopy (TEM) is typically used for investigating morphology which may be relevant in disease and can also be used for size. Recently, Cryo‐EM using liquid nitrogen for sample fixation which preserves EV morphology of EVs has been gaining attention as EV subpopulation morphology may change in disease, for example, EVs isolated from prion‐infected hippocampal neurons (GT1‐7) contained morphologies of double and triple membraned vesicles [154]. Diversity even among a single cell type has been shown among EVs isolated from human mast cell 1 (HMC‐1) conditioned media, which demonstrated nine total EV morphologies [153]. Morphological changes in EV subpopulations may therefore offer an alternative to characterization over size. Cryo‐EM can also be combined with immune gold labelling to provide insights into correlations between morphology and composition. Experiments in plasma investigating platelet activation and the subsequent population differences, finding CD41 expression in 60% of the population and CD63 expression in both S‐EVs and L‐EVs [50].

Together methods such as these can be utilized to understand EV subpopulations and their functionality. Current questions in the field, particularly for therapeutics, are based around finding and understanding the unique composition of the EV population responsible for the desired function. It is necessary as well to understand the number of EVs that are required to induce a response, in other words, potency, in addition to understanding the mechanisms involved. Determining the relevant EV subpopulation is critical, low uptake or cargo release may be due to differences in functional subpopulations. Particularly in vivo where it may be challenging to identify the origin of individual EVs and track populations without first knowing the exact EV composition.

The heterogeneity of EVs introduces a level of complexity in explaining and understanding the functions of EVs. Size, while a useful isolation method is not able to resolve this heterogeneity. Affinity‐based methods targeting known protein differences in EVs might offer a useful alternative and tool for dissecting the functions of EVs. This will in turn contribute to the further development of EV‐based therapeutics and EV‐based biomarkers for diagnostics.

## CONFLICT OF INTEREST

The authors declare no conflict of interest.
